# Hans Paulsen: Contributions to the Investigations of Glycoprotein Biosynthesis

**DOI:** 10.3390/molecules30183735

**Published:** 2025-09-14

**Authors:** Inka Brockhausen

**Affiliations:** Department of Biomedical and Molecular Sciences, Queen’s University, Kingston, ON K7L 3N6, Canada; brockhau@queensu.ca

**Keywords:** glycosyltransferases, glycoproteins, N-glycosylation, mucins, O-glycosylation, substrates, inhibitors, glycopeptides

## Abstract

Hans Paulsen was one of the first scientists who believed that chemistry should be applied to biology and medicine. His interest in natural products and their roles solidified in the 1970s. He passed on his knowledge to hundreds of students and coworkers and advanced science with many national and international collaborators. No matter where he was, at home or travelling, he was always curious and keen to learn, from chemistry to enzymes, their roles in diseases, and the possible applications of synthetic compounds. His creative chemistry and synthesis of novel compounds made essential contributions to elucidating the mechanisms and pathways of glycoprotein biosynthesis. This review describes the biosynthetic pathways of the O- and N-glycans of glycoproteins and studies of novel substrates and inhibitors developed by Hans Paulsen’s group.

## 1. Introduction

Hans Paulsen was a highly respected and successful carbohydrate chemist, valued and admired by his many coworkers, students, and collaborators. He was extremely knowledgeable in chemistry and science, but also in other cultures, politics, and history. Hans Paulsen was kind and compassionate and always interested until his passing in 2024 at the age of 102. His major strengths included an incredible memory and his ability to give informative and humorous lectures. When he developed a research plan, his excitement was contagious, and his brilliance stimulated his students to do well. He was one of the first scientists who believed that chemistry should be applied to biology and medicine. His interest in natural products and their roles solidified in the 1970s. He passed on his knowledge to hundreds of students and coworkers, and advanced science with many national and international collaborators. Paulsen visited many countries and studied their cultures and history. He enjoyed visits to Toronto, Amherst Island, and the Northern wilderness of Canada. The natural environment was conducive to making plans and discussing the value of current and future experiments and approaches to solving biochemical problems. The wilderness did not distract him from learning about enzymes, their roles in diseases, and their possible applications in solving problems through biochemistry. Always curious and full of humour, politeness, and modesty, he was a real pleasure to collaborate with.

Knowledge of the enzymes and mechanisms involved in transferring sugar residues to proteins was minimal in the 1980s. Hans Paulsen’s tremendous efforts and many contributions were critical for the advancement in the field of glycoprotein biosynthesis. Accounts of Hans Paulsen’s long and successful life as a carbohydrate chemist professor and emeritus have been published in journals and symposia. His main interests were carbohydrate chemistry, synthesis of unusual carbohydrate residues and linkages, selective oxidation, and specific modifications and substitutions of carbohydrate derivatives. This led to many glycan derivatives that proved to be valuable in determining enzyme specificities for their carbohydrate substrates and biosynthetic pathways.

Multiple novel chemical reactions were developed by Paulsen that established the groundwork for further chemistry of carbohydrates. While working with Kurt Heyns, Paulsen accomplished the catalytic oxidation of unusual carbohydrates [1]. He studied the role of sugar configuration and conformation in catalysis using platinum contact [2]. A major effort was made in the synthesis of carbohydrates containing nitrogen in the ring and their conformational analyses by NMR [3,4]. Many of these compounds were later shown to be potent glycosidase inhibitors [5]. Conformational analyses by NMR were developed and became standard analyses for carbohydrates [6]. He contributed to an understanding of the exo-anomeric effect and the dynamics of carbohydrate conformations. The chemistry of acyloxonium ion rearrangements was applied to many different compounds [7,8], including phosphate-containing carbohydrates [9]. These studies were extended to the synthesis and analysis of glycoprotein fragments [10]. Carbohydrates were analyzed in solution and in crystal form [11,12], contributing to our knowledge of carbohydrate structures and their biological roles.

Paulsen’s efforts to advance the analyses of carbohydrate conformations were helpful in delineating the substrate binding of glycosyltransferases (GTs) and in designing specific substrate analog inhibitors. He also performed analyses of natural products, which turned out to be glucosidase and mannosidase inhibitors. These compounds could be valuable for therapy development in diseases associated with alterations of specific glycans or for controlling the immune system that relies on glycan recognition. The advancements in the structural analysis of glycans were crucial to the success of synthetic chemistry and its application in glycobiology and medicine.

The intensive efforts of the Paulsen lab succeeded in the synthesis of specific glycan derivatives for studies of glycobiology and the role of glycoproteins in cancer and immunology. Many other results of Paulsen’s research led to knowledge of bacterial lipopolysaccharides and the production of specific antibodies and glycoprotein antigens. Thus, Paulsen’s ideas and efforts in chemical synthesis and refined methods of structural analysis advanced our knowledge, especially in cancer cell biology. The wealth of synthetic compounds led to new knowledge of glycosyltransferase (GT) specificity, pathways, and glycoprotein functions. Compounds representing specific GT substrates were synthesized [13], allowing assays of enzyme preparation from cells or tissues expressing multiple GTs [14].

This review describes the biosynthesis of N-glycans and mucin type O-glycans by human enzymes and summarizes the work done by the Paulsen group to use synthetic chemistry for the acquisition of knowledge of biosynthetic enzymes and insight into the role of glycoproteins in health and disease.


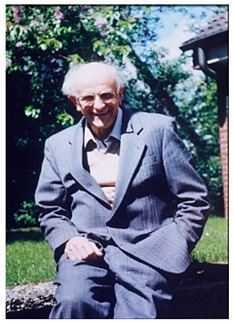


## 2. Glycosyltransferases

The enzymes that build complex sugar chains of glycoproteins are glycosyltransferases (GTs), which have been classified into 138 families by the CAZy database (as of July 2025), based on their amino acid sequence, predicted 3-dimensional fold, mechanism, and known activity. Inverting GTs inverts the anomeric configuration of the sugar in the donor substrate to form the opposite linkage in the reaction product, while retaining GTs retain this linkage. The nucleotide sugar donor substrates for glycoproteins are UDP-αGlcNAc, UDP-αGal, UDP-αGalNAc, GDP-αMan, GDP-βFuc and CMP-βSialic acid. Dolichol (Dol) is used for membrane-bound intermediates in the endoplasmic reticulum (ER)—in particular, Dol-P-Manβ and Dol-P-Glcβ. The GT protein folds are GT-A, GT-B, and GT-C (CAZy), which have been confirmed with a limited number of crystal structures (Table 1). Table 1 lists the major GTs involved in the biosynthesis of N-glycans and mucin-type O-glycans.

Some of these GTs have broad specificities and thus can extend a number of glycoconjugates, including glycolipids and/or proteoglycans. Many GTs occur as natural variants that may have slightly different properties and specificities. Only one GT (C1GALT1) requires the coexpression of a chaperone (COSMC). Often, GTs form dimers or multi-enzyme complexes that support activities. GTs requiring Dol intermediates are localized to the membranes of the ER. The initial N-glycan structures up to Man_5_GlcNAc_2_- are made on the cytoplasmic side of the ER membrane, and after being flipped to the lumenal side, N-glycans are completed and transferred from Dol to nascent peptide. Further N-glycan processing and extension reactions occur after the transport of the glycoprotein to the Golgi.

GTA-folded GTs have one major fold with a central catalytic domain. GT-B folded GTs have 2 typical Rossman-like (nucleotide-binding) folds and the active site located in between them. GT-C folded GTs are uncommon, and Dol-binding GTs often have multiple membrane-spanning domains that are typical of GT-C folded enzymes [15]. The enzyme mechanisms of a few GTs have been suggested by their crystal structures, analysis of mutants, and enzyme kinetics. Many GTs are N-linked glycoproteins and can also be O-glycosylated.

In contrast to GTs, glycoside hydrolases are classified into 194 families (as of July 2025), with many subfamilies. These enzymes hydrolyze a glycosidic bond using a catalytic acid as proton donor and nucleophilic catalytic base. Like GTs, hydrolases act with an inverting or retaining mechanism and have a limited number of protein folds.

Most inverting GTs appear to follow a single displacement mechanism where a specific hydroxyl of the acceptor substrate forms a nucleophile that attacks carbon-1 of the sugar linked to phosphate of the nucleotide sugar donor substrate. This often involves a catalytic Asp residue in a DxD sequence [16]. The mechanisms of retaining GTs where sugar linkages in donor and product are the same remain to be clarified further. In the Golgi, GTs are commonly type II membrane proteins with a short N-terminal cytoplasmic domain followed by a transmembrane ™ domain, and the folded GT domain in the lumen of the Golgi.

GlcNAc-transferases (GnT, MGAT) that synthesize N-glycan antennae are highly specific for the antennae they synthesize [17]. This specificity is likely a result of the large internal substrate-binding cavity that accommodates more than the terminal glycan. This is in contrast to certain Gal-transferases (GalTs) that can act on a single sugar residue. For example, the β1,4-GalT B4GALT1 only requires GlcNAc as a substrate, although some branch specificity for N-glycan acceptors has been observed, which may be due to steric factors [18,19]. Acceptor substrates have been designed that have all of the structures required for recognition by a specific enzyme but are as small as possible, conveniently synthesized, and handled by enzyme assays. GTs are usually highly specific for their donor substrates, with small modifications of the sugar moiety being tolerated and allowing a modified sugar to be transferred.

## 3. Measurements of Glycosyltransferase Activities

The general GT reaction is Acceptor + Donor → Product + Nucleotide. The reaction often requires a divalent metal ion to complex the nucleotide (e.g., Mn^2+^ or Mg^2+^), along with a buffer and detergent if a membrane-bound version of GT is used. If the enzyme source has contaminating hydrolases, specific inhibitors can increase the product yield. The GT activities can be measured accurately by tracking the transfer of a radioactive sugar residue to form the reaction product. When neutral acceptor substrates are used, the radioactive nucleotide sugar and sugar-phosphate breakdown products can be separated after incubation by anion exchange chromatography. Scintillation counting of the reaction product in the flow-through fractions will indicate the exact amount of sugar transferred, allowing the specific enzyme activity and kinetics to be determined. Most of Paulsen’s compounds have been examined using the sensitive radioactive method.

If charged acceptors are used, then other assay methods could include hydrophobic chromatography, HPLC, electrophoresis, or paper chromatography. Assays using non-radioactive donor substrates require HPLC separation of assay components and mass spectrometry (MS) for estimating the product yield. TLC can also be used with a panel of standard compounds. Another method is to detect the reaction product UDP by bioluminescence (UDP-Glo ^TM^). In order to determine the linkage synthesized, specific hydrolases, antibodies, or lectins could be employed. If sufficient amounts of reaction product are available, NMR is the best method for determining anomeric configuration and sugar linkage in the product.

For accurate and efficient measurements of GT activities, it is crucial to design a suitable acceptor substrate and select the best method to determine the amount and the new linkage in the reaction products. Paulsen’s N-glycan derivatives with an octyl aglycone group were ideal for isolating reaction products by columns of silica-linked hydrophobic chains of 18 carbons. By omitting specific sugar residues, the minimum size of the active acceptor could be assessed. For GTs acting on O-glycans, aryl aglycone groups have been successful in both anion exchange and hydrophobic chromatography assays. Glycopeptide acceptors were used mainly by anion exchange chromatography and HPLC.

## 4. Biological Significance of Glycoproteins

Most eukaryotic proteins are post-translationally modified with glycans and are found inside cells, on cell surfaces, and in secretions. The Asn-linked N-glycan and Ser/Thr-linked mucin-type O-glycan structures in human glycoproteins contain the same basic sugar residues, except that these O-glycans do not have Glc and Man residues. Many of the internal and terminal carbohydrate epitopes of extended glycan structures are similar. The N- and O-glycans of glycoproteins serve to provide protein stability, conformation, folding, intracellular targeting, resistance to degradation, and dehydration. Protein glycosylation is involved in many biological functions, in the immune system, during development and fertilization, and as lubricant. The glycan moieties can interact with microbes and protect against the invasion of pathogens. Lectins and antibodies bind to glycans, which play important roles in cell–cell and cell surface receptors interactions.

## 5. Glycoprotein Biosynthesis

**Figure 1:**
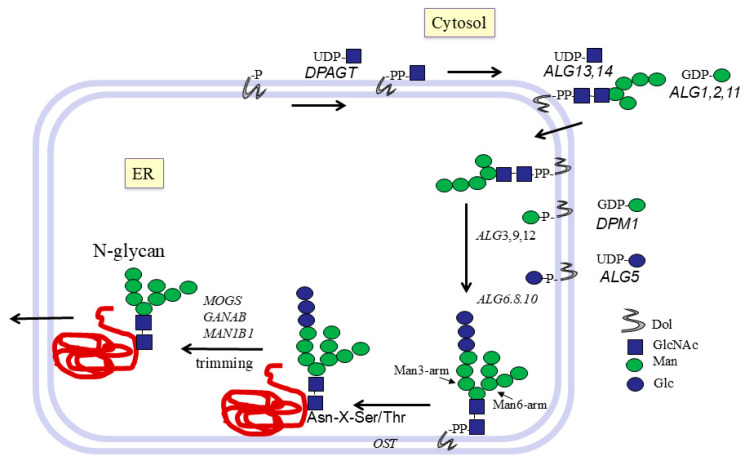
Early pathways of N-glycan biosynthesis in the ER.

The initial pathways of N-glycan synthesis involve Dol lipid carriers at the cytoplasmic and lumenal sides of the ER membrane. Preformed oligosaccharides are transferred from Dol to peptides in the ER (Figure 1). In contrast, O-glycans are all added to the protein in the Golgi and do not involve Dol. During N-glycan processing, Glc and Man are removed by specific hydrolases, a process not known for O-glycan synthesis. Sulfotransferases can add sulfate esters in the Golgi to Gal or GlcNAc residues of both N- and O-glycans by transferring sulfate from 3′-phosphoadenosine-5′-phosphosulfate.

Paulsen’s work has contributed immensely to our knowledge of specific enzymes and pathways. The substrate binding and catalytic mechanisms of the enzymes identified in this work may differ between homologues from different species and tissues, and many enzyme variants occur. Nature has provided human mutations that unfortunately result in disease and suffering but has helped the biochemist to identify enzyme functions and the necessity for protein glycosylation. Thus, chemistry also played an important role in the discovery of the significance of glycosylation in disease.

Mucin glycoproteins have a high content of mucin-type GalNAcα-Ser/Thr-based O-glycans. Although many alterations of O-glycans in disease have been identified, there are no human mutants that lack mucin O-glycans, and 20 or more polypeptide GalNAc-transferases (GALNTs) cooperate in humans to assemble these O-glycans, indicating that they are essential for survival. The O-glycosylation of non-mucin proteins depends on the protein folding and exposure of Ser/Thr residues as potential O-glycosylation sites.

There are a number of other O-glycans that are important, e.g., O-αMan glycans of dystroglycans that may be altered in muscular dystrophies. O-αFuc is found in signalling proteins, and O-βGal in collagens. O-GlcNAcβ-Ser/Thr (O-GlcNAc), along with phosphate modifications, is common for cytoplasmic and nuclear proteins and functions in a wide range of biological activities, from apoptosis to oxidative stress responses. O-GlcNAc is not extended by additional sugars in the Golgi. However, it can be modified in vitro by β1,4-GalT. Alterations of O-GlcNAc have been implicated in protein aggregation, neurological diseases, cancer, and diabetes.

## 6. N-Glycans

**Figure 2:**
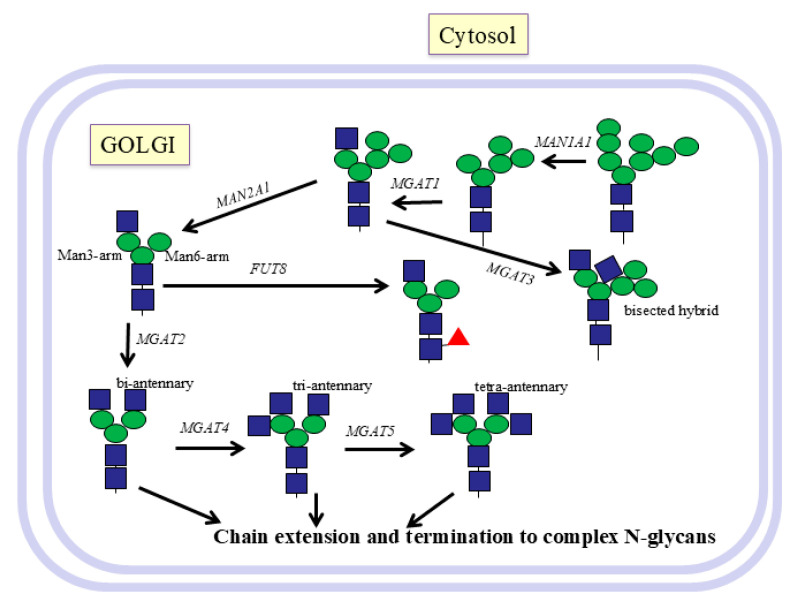
Golgi pathways of N-glycosylation. N-glycans are processed to hybrid or complex N-glycans and extension of the antennae in the Golgi. Glycans are trimmed by mannosidase, followed by the transfer of GlcNAc in β1-2 linkage to Manα1-3 of the Man3-arm by MGAT1. This facilitates further synthesis by α6-fucosylation of core GlcNAc attached to Asn, trimming of Man residues on the Man6-arm, and synthesis of further antennae by MGAT2,4,5 and bisecting structures by MGAT3. The antennae are then extended by a wealth of different enzymes that introduce repeating Gal-GlcNAc units and many possible branched and linear epitopes, Lewis antigens, blood groups, and tissue-specific structures. Glycoproteins are then transported to their preferred locations.

N-glycans are found in all animals, as well as in many other species, and even in archaea and certain bacteria. Viruses usually carry glycoproteins with glycans that are characteristic of the cell type the virus originates from. High-mannose N-glycans have only Man residues extending the Man_3_GlcNAc_2_ core and are the least processed chains. Hybrid N-glycans have several Man residues on the Man6 arm and a GlcNAcβ1-2 antenna on the Man residue of the Man3 arm (Figure 2).

The antennae of complex N-glycans often consist of repeating Galβ1-4GlcNAcβ1-3- (LacNAc) sequences that can carry many possible internal and terminal sugars and epitopes, such as GlcNAc, Gal, GalNAc, Fuc, sialic acid (Sia), blood groups, i and I antigens, and LacdiNAc and Lewis determinants (Table 2). Many of these are shared with complex O-glycans and glycosphingolipids. The aberrant synthesis of N-glycans and O-glycans, leading to altered amounts of epitopes in cancer, is controlled by many enzymes. The enzymatic basis for these changes is extremely complex and usually involves the altered expression of a number of enzymes involved in the glycosylation pathways.

N-glycans have gained considerable interest due to their multiple functions in protein structure, biology, and medicine. The functionally important N-glycan antennae cover a significant amount of space around glycoproteins and can be recognized by antibodies or lectins and carbohydrate-binding proteins of the immune system. N-glycan structures are often altered in cancer and in patients with congenital disorders of glycosylation (CDG), which can affect the properties and essential functions of glycoproteins [32]. For example, in cancer cells, GnT V (Table 1, Figure 2) was shown to increase in activity, thus expanding the size and complexity of branched tetra-antennary N-glycans. Thus, our focus was on designing inhibitors for N-glycan branching enzymes and controlling the functions of N-glycans.

The pathways of N-glycan synthesis are highly complex, involving many highly conserved enzymes in the ER, Golgi, cytoplasmic, and nuclear compartments and various mechanisms of transport and localization. In the early 1980s, Hans Paulsen started a fruitful collaboration with Harry Schachter (University of Toronto) to elucidate the substrate specificities and control of N-glycosylation pathways. For this purpose, Paulsen synthesized a series of N-glycans with systematic modifications of hydroxyl groups in order to explore their importance in GnT activities. Compounds included deoxy derivatives or those with specific reactive or non-reactive modifications and linkers. N-glycans were synthesized as reducing sugars or with a hydrophobic aglycone group that was useful for enzymatic reactions and isolation of enzyme products by HPLC. These derivatives helped determine the detailed recognition of substrates and enzyme specificities. Depending on the enzyme, specific hydroxyl groups were found to be critically required, and modifications in the stereochemistry, bulkiness, and hydrophobicity of substituents had variable effects on activity. These studies were complemented by our understanding of protein structures, their proposed mechanism, and the amino acids controlling the active site [33].

## 7. Early Pathways of N-Glycosylation at ER Membranes

N-glycosylation involves both nucleotide sugars and Dol-P-sugars. The early pathways to the Man_5_GlcNAc_2_-PP-dolichol structure are catalyzed by enzymes in the ER membrane. Reactions require UDP-GlcNAc and GDP-Man nucleotide sugars present in the cytosol. The N-glycan is then flipped across the membrane for further processing. In order to synthesize N-glycans, the cell must provide nucleotide sugar donor substrates and acceptor substrates that are Dol-P intermediates for the initial pathways (Figure 1) localized to the ER membrane. These early reactions have been studied mainly in yeast [34]. The biochemical importance of several of these enzymes was identified in humans with gene mutations leading to the CDG syndrome. Due to the reduction or absence of specific activities involved in the assembly of N-glycan structures, glycoproteins lack N-glycans and exhibit different electrophoretic mobility. This defect can be used as a diagnostic marker for CDG.

The initial cytoplasmic reaction is the transfer of GlcNAcα-P from UDP-GlcNAc to Dol-P by GlcNAc-1-P-transferase, named ALG7, in yeast, to form Dol-PP-αGlcNAc (Figure 1). UDP-GlcNAc: Dol-P GlcNAc phosphotransferase DPAGT1 binds to the UDP-GlcNAc analog antibiotic tunicamycin [35]. Tunicamycin also binds to GlcNAc-1-P transferases from other species, including bacteria.

An oligosaccharide consisting of 2 GlcNAc and 5 Man residues is assembled on Dol-P from UDP-αGlcNAc and GDP-αMan on the cytoplasmic side of the ER membrane. The GnT complex ALG13/ALG14 first synthesizes the di-N-acetyl-chitobiose structure GlcNAcβ1-4GlcNAc- [36]. Subsequently, the Manβ1-4 linkage to GlcNAc is synthesized by β1,4-Man-transferase (ManT) ALG1 (Table 1). ALG2 transfers Manα3 as well as Manα6 (forming the Man3- and Man6-arms) from GDP-Man to the Manβ1-4 residue. Two Man residues are then added in α1-2 linkage to the Man6 arm by α2ManT ALG11. The Dol-PP-linked Man_5_GlcNAc_2_ oligosaccharide is then transferred to the ER lumen for the further addition of Man and Glc residues. Dol is a large membrane lipid that has a significant effect on the structure and leakiness of the membrane and may be involved in the translocation process of the Man_5_-GlcNAc_2_ intermediate. The enzyme that transfers Man to Dol-P has been shown to require membrane phospholipids for its activity [37].

For the reactions taking place at the lumenal side of the ER membrane, both acceptor and donor substrates utilize membrane-bound Dol-P sugars that flip across the ER membrane. The donor substrate Dol-P-βMan is synthesized at the cytoplasmic side from Dol-P and GDP-Man by Dol-P-Man synthase DPM1. Dol-P-βGlc is synthesized by Dol-P-Glc synthase ALG5 using UDP-αGlc. The Glc and Man residues of these membrane-bound donor substrates are then translocated to the lumen of the ER, where they are used by GTs to transfer Man and Glc to Man_5_-GlcNAc_2_-PP-Dol to synthesize the large Glc_3_-Man_9_-GlcNAc_2_ structure (Figure 2). The transfer of Man residues involves α3ManT ALG3 and α2ManTs ALG9 and ALG12. The final Glc residues are transferred to the Man3-arm by α3Glc-transferase (GlcT) ALG6 and ALG8, and the terminal Glc residue is transferred by α2GlcT ALG10 (Table 1).

## 8. Transfer of N-Glycans from Dolichol to Protein

After the large oligosaccharide has been assembled, it is transferred from Dol-PP to the nitrogen side chain of Asn of the nascent glycoprotein in the ER by the oligosaccharyltransferase complex OST. This inverts the α-linkage of the first GlcNAc to a β-linkage. The Asn-x-Ser/Thr sequon is absolutely required for protein N-glycosylation, and this requirement has been maintained in all eukaryotic species throughout evolution, including in archaea and several bacteria. A Pro residue between Asn and Ser/Thr or following Ser/Thr does not allow N-glycosylation, likely by preventing the formation of an essential hydrogen bond between Asn and Ser/Thr.

Processing reactions start in the ER lumen after the attachment of N-glycans to peptides. A number of chaperone and GlcT proteins are involved in protein folding and quality control. The terminal Glcα1-2 residue is cleaved by glucosidase MOGS, followed by cleavage of two Glcα1-3 residues by GANAB. Mannosidase MAN1B1 then cleaves Manα1-2, leaving a mixture of N-glycan structures from Man_8_-GlcNAc_2_ to Man_5_-GlcNAc_2_, which are precursors of hybrid and complex N-glycans. These glycoside hydrolases are type II membrane proteins, but are classified into different CAZy GH families (Table 1). The glycoproteins are then transported from the ER to the Golgi, where further glycan processing and synthesis occurs.

## 9. Golgi Reactions

All GTs in the Golgi utilize nucleotide sugar donor substrates that need to be imported into the Golgi by specific transporters. The localization of Golgi proteins is due to the gradual transport of membranous vesicles through Golgi compartments. The relatively short tm domain of GTs does not appear to be important for activity, and recombinant GTs produced in vitro usually lack the tm domain to enhance solubility. However, it is possible that the short N-terminal cytoplasmic tail of GTs plays a role in the localization of GTs within various Golgi compartments. The conserved oligomeric Golgi protein complex (COG) has been shown to control GT localization as well as retrograde Golgi to ER transport. In addition to the ER proteins, the Golgi resident protein QSOX1 also catalyzes the disulfide bonding of specific GTs, e.g., sialyltransferases (SiaTs), as well as the intermolecular bonds of large mucins [38]. However, the complex mechanisms that localize GTs and re-localize them under pathological conditions remain to be further explored. Processed and extended glycoproteins are then delivered to their final destination on the cell surface, in secretions, or in intracellular membranes.

## 10. Synthesis of N-Glycan Antennae by GlcNAc-Transferases

After transport of the glycoprotein from the ER to the Golgi, further Man residues are cleaved from N-glycans by mannosidases that are type II membrane proteins. Mannosidase 1 MAN1A1 progressively cleaves Manα1-2 residues. MGAT1 (GnT I) is the first enzyme to add an antenna and transfers GlcNAc in β1-2 linkage from UDP-GlcNAc to the Manα1-3 residue (Man3-arm) of Man_5_-GlcNAc_2_-Asn-R (Figure 2). The conversion of hybrid to complex N-glycans is then controlled by mannosidase 2, MAN2A1, which cleaves both the Manα1-3 and Manα1-6 residues from Manα1-6 (Man6-arm), which then exposes the substrate for GnT II.

Harry Schachter, together with Pamela Stanley [39], discovered GnT I in mutant CHO cells. It was later shown that this enzyme is essential for further modifications of N-glycans. This includes the removal of 2 Man residues by Golgi mannosidase 2, α6-fucosylation of the core GlcNAc linked to Asn, and further branching by GnT II (MGAT2) and IV (MGAT4), which then allows the formation of the fourth antenna by GnT V (MGAT5) (Figure 2). MGAT1 is also required for introducing the bisecting GlcNAc by GnT III (MGAT3) [32].

## 11. GnT I-MGAT1

The characterization of GnT enzymes was greatly facilitated by collaboration with Hans Paulsen. Paulsen’s group was the first to synthesize N-glycan oligosaccharides having the Man β1-4GlcNAc linkage. The configuration and rotation of the N-acetyl group of GlcNAc represented a steric hindrance that favoured the synthesis of Manα-glycosides. Thus, the method for Manβ- synthesis was a breakthrough for the synthesis of GnT substrates of N-glycans up to 9 residues [40]. Glycan linkages and 3D conformations were extensively analyzed by NMR [41].

As an effective acceptor substrate for GnT I [39] that adds GlcNAc to the Man3-arm, Paulsen’s group synthesized Manα6(Manα3)Manβ-octyl and many related compounds. It was shown that the minimum requirement for an acceptor substrate for purified rat liver GnT I is Manα1-3Manβ1-4GlcNAc, but optimal activity is observed when the Manα1-6 residue is also present. Other groups, e.g., Ole Hindsgaul (University of Alberta), Jeremy Carver (University of Toronto), and Khushi Matta (Roswell Park Institute, Buffalo, NY), also participated in the synthesis of acceptor substrates for GnTs with hydrophobic groups replacing the di-N-acetylchitobiose of the N-glycan core and helped to successfully characterize properties of GnTs.

The crystal structure of rabbit GnT I suggested its catalytic mechanism as an inverting GT-A-folded enzyme that binds UDP-GlcNAc and Mn^2+^ [33] (Table 1). The protein has a ^211^EDD^213^ motif involved in forming hydrogen bonds and interacting with UDP-GlcNAc and Mn^2+^. D291 was proposed to be the catalytic base that can deprotonate the 2-hydroxyl of Manα1-3 in the acceptor, which then becomes a nucleophile that attacks carbon-1 of GlcNAc in the donor UDP-GlcNAc. The proposed acceptor binding site was shown by Manα1-3 modeled into the GnT I crystal structure, which is consistent with the specificity results using Paulsen’s compounds.

Paulsen’s group made significant efforts in replacing hydroxyls of the Manα1-3(Manα1-6)Manβ-octyl acceptor for GnT I with a number of small and large substituents, including O-methyl, O-propyl, O-pentyl, 4-pentenyl, 4-pentanolyl, 4-oxo-pentyl, 4,4-azo-pentyl (diazirino-pentyl), 4,5-epoxy-pentyl, 5-amino-pentyl, 5-pentanolyl, 5-iodoacetamido-pentyl. In other derivatives, the oxygen was removed or replaced with NH_3_ [42]. The deoxy compounds indicated the requirement of the hydroxyl group, possibly as a hydrogen bond donor, while loss of activity after replacement of hydroxyl groups with larger substituents may be a result of steric hindrance.

After this extended production of new compounds, GnT I assays showed that the 2,3 and 6-hydroxyls of the Manα1-3 residue as well as the 2- and 4-hydroxyl of the Manβ residue were required for activity. The N-acetylchitobiose linked to Asn-peptide was not required and could be replaced by a number of different aglycones, including GlcNAc, hexyl, octyl, or other hydrophobic groups [14,17]. Although the Manα1-6 residue was required for optimal activity, removal of its 2-hydroxyl or replacement of the 3-, 4-, or 6-hydroxyl with an O-methyl group still allowed GnT I activity at various levels. These compounds helped to explore the substrate specificity of GnT I and the enzymatic production of acceptor substrates for the next GnTs in the pathway. This approach was also part of the design of specific competitive or irreversible inhibitors. A number of these derivatives were inhibitors with *K*_i_ values between 0.76 and 10.2 mM. The most potent inhibitor was Manα6(6-O-methyl-Manα3)Manβ-octyl, where presumably the methyl group clashed with binding of the substrate to the active site of GnT I (Table 3).

## 12. GlcNAc-Transferases (MGAT2-6)

After GnT I has acted, GnT II synthesizes the second antenna by transferring a GlcNAc residue in β1-2 linkage to the Manα1-6 residue (Figure 2). The serious human condition of CDG IIa is due to a mutation in GnT II. The synthesis of a large series of substrate analogs of the standard GnT II acceptor Manα1-6(GlcNAcβ1-2Manα1-3)Manβ-octyl allowed determination of the specificity of partially purified rat liver GnT II [43,44]. GnT II requires the 3-hydroxyl of the GlcNAc added by GnT I on the Man3-arm as well as the 3-hydroxyl of the Manα1-3 residue, which is essential for binding to GnT II. Interestingly, the trisaccharide GlcNAcβ1-2Manα1-3Manβ-octyl that lacks the Manα1-6 residue is not a substrate but is a good inhibitor of GnT II with a K_i_ of 0.9 mM (Table 3).

Removal of the 2-hydroxyl of Manα1-6, the site of GlcNAc addition, creates a competitive inhibitor for GnT II with a K_i_ of 0.13 mM, while replacement by a methyl group eliminates activity and does not inhibit, likely because of steric hindrance [45]. In contrast, the 3-, 4-, and 6-hydroxyls of Manα1-6 are not required for activity. In contrast to GnT I, GnT II does not need to interact with the 4-hydroxyl of Manβ-. However, substitution of the 4-hydroxyl of Manβ- by a methyl group blocked GnT II activity. This may explain why GnT II cannot act after GnT III, which introduces a bisecting GlcNAcβ1-4 residue linked to Manβ [43,44].

The structure of human MGAT2 was determined in complex with Mn^2+^/UDP-GlcNAc or with the donor analog UDP and with the acceptor GlcNAcβ1-2Manα1-3(Manα1-6)Manβ1-4GlcNAc_2_-Asn [46]. This suggested that the inverting GnT II has an overall fold resembling GT-A and that D347 is the catalytic base residue.

After the second antenna has been established, GnT IV and V can add additional GlcNAc residues that initiate tri- and tetra-antennary N-glycans, respectively (Figure 2).

GnT IV from the hen oviduct was shown to be a β1,4-GnT that can act on glycopeptides with the bi-antennary structure GlcNAcβ1-2Manα1-3 (GlcNAcβ1-2Manα1-6) Manβ1-4GlcNAc1-4GlcNAc-Asn- where both Man3- and Man6-arms had to have GlcNAc residues (added by GnT I and II) for full activity. Extension of these GlcNAc residues by Gal yielded 5% activity, while sialylation completely blocked GnT IV activity.

**Figure 3:**
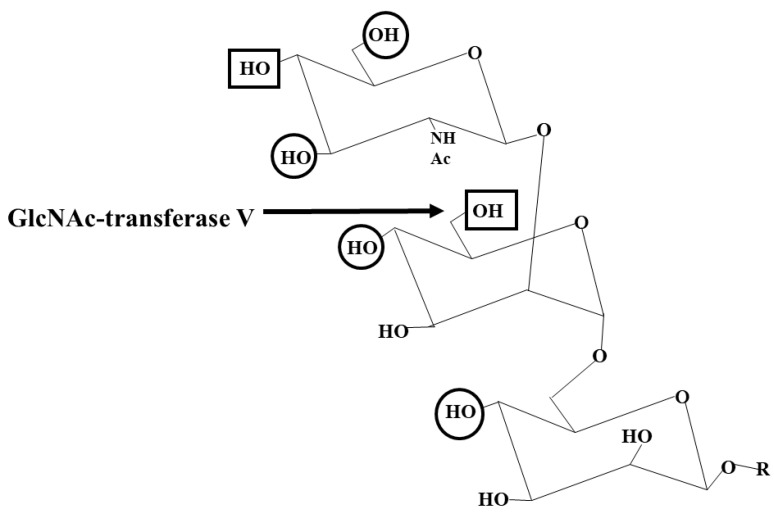
Specificity of GlcNAc-transferase V. GnT V transfers a GlcNAcβ residue to the 6-hydroxyl of the Manα1-6 residue of the N-glycan core. The acceptor substrate for purified GnT V from the hamster kidney was a derivative of the 6-arm, GlcNAcβ1-2Manα1-6Manβ-R. The 3-arm of the N-glycans is not essential for GnT V activity. R can be an oligosaccharide or a hydrophobic group such as octyl. The Manβ-residue can be replaced by Glcβ having full activity. To determine acceptor specificity, hydroxyls were deleted or replaced by a larger group. The rectangular boxes indicate an absolute requirement for the hydroxyl, while the circles indicate that the hydroxyl is important for the activity. Replacement of the 4- and 6-hydroxyls of the Manα1-6 residue with an O-methyl group yielded the GnT V inhibitor GlcNAcβ1-2(4,6-di-O-methyl)Manαl-6Glcβ-*p*np.

The tetra-antennary N-glycans are synthesized by GnT V, which is involved in cell adhesion and recognition and in cancer development [47] (Table 2). Thus, GnT V inhibitors are of importance to obtain potential tools to fight cancer and metastasis (Table 3). In contrast to GnT II, GnT V from the hamster kidney, which also acts on the Man6-arm, does not require the Man3-arm in its acceptor substrate and can act on linear trisaccharides such as GlcNAcβ1-2Manα1-6Manβ-octyl [48,49]. Paulsen’s group synthesized biantennary substrate analogs that showed how specific hydroxyls of both the Man3- and Man6-arms influence GnT V activity (Figure 3). However, while the 4-hydroxyl of GlcNAcβ1-2 and the 4- and 6-hydroxyls of Manα1-6 on the Man6-arm were absolutely essential for activity, replacement of the 3-hydroxyl of GlcNAcβ1-2 with a larger pivaloyl group prevented activity, presumably due to steric interference [48]. Kanie et al. [50,51] also reported the critical role of the 3-, 4-, and 6-hydroxyls of the β1-2-linked GlcNAc in enzyme recognition. This clearly established the pathway of biosynthesis, where GnT II must add a GlcNAcβ1-2 residue to the Man6-arm before GnT V can act, and transfer of Galβ1-4 to GlcNAcβ1-2 blocks GnT V action.

GnT V is an inverting GT, but does not appear to have a DxD motif. Instead, a Glu residue forms the catalytic base, and the activity is independent of divalent metal ions. The crystal structure of the lumenal domain shows a GT-B-like fold with 2 Rossmann domains [52]. A pocket that accommodates the Man1-6 residue of the acceptor is lined by the aromatic rings of F380 and W401. GnT V requires a non-catalytic domain for activity towards glycoproteins [53,54].

GnT III requires the prior action of GnT I to add the bisecting GlcNAcβ1-4 to the internal core Manβ1-4 residue. The enzyme can also act on biantennary substrates and produce hybrid and biantennary bisected N-glycans, which causes a crowded configuration that may be the reason for the reduction of further N-glycan antennae synthesis and processing. Paulsen synthesized N-glycans with 9 sugar residues, including the bisecting GlcNAc [40]. The steric effect of the bisecting GlcNAc was examined by NMR, which showed that the Manα1-6 residue is present in a different (gg) preferred conformation [55].

GnT VI synthesizes an additional antenna on the Man6-arm. This β1,4-GnT activity was found in hen oviduct [56,57] and fish. GnT VI can act on the trisaccharide acceptor GlcNAcβ1-2 (GlcNAcβ1-4)Manα-methyl, which resembles the product of GnT II and GnT V.

## 13. Modifications of N-Glycan Antennae

Lysosomal hydrolases have N-glycans with a specific Man-6-phosphate (Man6-P) marker that targets them to the lysosomes via a specific Man6-P receptor [58]. In Golgi, the GlcNAc-P-transferase GNPT ABG complex transfers GlcNAc-P from UDP-GlcNAc to the 6-hydroxyl of a Man residue on lysosomal enzymes. This is followed by hydrolysis of GlcNAc to expose the Man6-P marker that can bind to the Man6-P receptor in the Golgi, which reroutes the glycoprotein to acidic compartments and to the lysosomes.

Paulsen’s compounds helped to define the association of glycoprotein biosynthesis in biological processes, such as apoptosis in porcine aortic endothelial cells [59]. GnTs I and II are usually the major GnT activities in tissues and cultured cells, with GnTs III, IV, and V activities being low in vitro. Further extension of N-glycan antennae includes the addition of Gal by β1,4-GalT B4GalT1 [18]. The inhibition of Gal extension could reduce the addition of Sia and multiple other epitopes that may play a role in the immune system and in cancer [22] (Table 3). Sialic acids, which are often found in increased amounts in cancer, form selectin (Siglec) ligands and play a role in cancer cell metastasis, immune evasion, and cancer cell survival. The expression of ST6Gal1 has been shown to be increased in cancer [24]. ST6Gal1 has been crystalized with CMP. The inverting enzyme has a GT-A-like fold and may act via an S_N_2 mechanism [60]. However, much of the knowledge about SiaT mechanisms comes from studies of bacterial enzymes with similar activities.

## 14. Inhibition of Glycosyltransferases

GTs bind their substrates by hydrogen bonding and hydrophobic interactions and prefer specific conformations and flexibilities. Inhibitors may compete with substrates or covalently bind to GTs to cause loss of activity. Paulsen’s approach to inhibitor design was to synthesize many short and long analogs of natural acceptor substrates for GTs. The synthesis of potential inhibitors included removal of hydroxyls that are essential for sugar transfer, blocking access to critical hydroxyls by introducing large groups, or preventing the folding of enzymes in the active conformation. Other strategies that could cause loss of activity could focus on the donor binding site and the binding of essential divalent metal ions.

Compounds were either free oligosaccharides or had a hydrophobic group at the reducing end of the chain. Hydroxyl groups were either deleted or replaced by methyl groups or by potentially reactive substituents such as azide and epoxide groups that can react with nucleophilic amino acids, as well as diazirino and iodoacetamido groups that are activated by UV. These modified substrates enhanced our understanding of how GTs bind to their substrates, and that small changes in the stereochemistry, conformation, or electronic, bulky, or hydrophobic character of the sugar ring substituents, depending on their positions, could block enzyme activity by affecting interactions with specific amino acids in the substrate binding and catalytic sites of enzymes. A basic requirement for understanding biosynthetic mechanisms is knowledge of the enzyme structure and substrate specificity.

Several N-glycan derivatives proved to be potent inhibitors of GTs (Table 3). However, due to their large size and hydrophilicity, it is difficult to envision their transport across the membranes to reach the GTs in the Golgi.

Since extended glycan chains carry many cancer-associated antigens, inhibition of glycoprotein extension may be useful for biological studies. Gal is added to the GlcNAc of the N-glycan antennae by a family of enzymes (Table 1). We examined the specificity and inhibition of purified bovine B4GALT1 with a large panel of acceptor substrate derivatives. While GlcNAcβ-Bn was the standard acceptor, GlcNAcα-Bn was only 5% active, and free GlcNAc showed 29% activity, indicating that it is mainly the β anomer that forms the substrate. Glycopeptide acceptors having GlcNAcβ1-3/6GalNAc-glycans were relatively less active than GlcNAcβ-Bn. Derivatives with the large 2-naphthyl as aglycone could not support activity, and most of these compounds inhibited the enzyme. 1-Thio-N-butyrylGlcNAcβ(2-naphthyl) inhibited B4GALT1 by 100% at 1 mM with a *K*_i_ of 0.01 mM [18]. However, bicyclic quinolinyl compounds (having nitrogen in the ring) were active substrates. This indicates that the overall structure of the acceptor and the conformation and electronic properties of the aglycone are important for enzyme recognition. The catalytic domain of B4GALT1 has two flexible loops that change from an open to a closed conformation upon binding of UDP-Gal and metal ions [61]. This creates an acceptor binding site and seems to be a mechanism common to several GTs. After the reaction, the oligosaccharide product is released, and the loops revert to the initial conformation, releasing UDP. Thus, additional strategies are required to make inhibitory compounds applicable in biological systems. Future studies will be seeking higher potency and specificity of inhibitors, targeting strategies, and examining the biological effects in cell cultures and in animals before development into human therapeutics.

## 15. Mucin Glycoproteins

Mucins are very large glycoproteins that are heavily O-glycosylated, sometimes with 90% carbohydrates. They may also carry several N-glycans. Mucins form a viscoelastic mucus layer over the epithelia and protect the host from environmental stresses, dehydration, and infections. Humans have 20 or more genes that encode mucins and many other proteins that have mucin-like O-glycosylated domains. Human mucin-type O-glycans all start with a GalNAc residue αlinked to Ser or Thr. Four core structures are common, with several less common structures such as core 5, GalNAcα1-3GalNAc- (Table 4) [23,30]. These core structures can be sialylated or extended with structures and epitopes similar to those found on complex N-glycans. The O-glycan structures often vary between cell types and in conditions such as inflammation or in cancer and are regio-specific in the intestine. Thus, the expression of genes encoding GTs is delicately regulated, although our knowledge in this field is still marginal [23].

Mucins are characterized by variable number of tandem repeat (VNTR) regions that are rich in Thr/Ser/Pro residues and carry most of the O-glycans. There are three types of mucins in humans: cell surface-bound mucins, small, secreted mucins, and large, gel-forming mucins. For each mucin, several variants have been identified. Small, secreted mucins (MUC7) are relatively soluble and found in the saliva. Cell surface-bound mucins (MUC1,3,4,12,13,15,16,17,20,21,22) have a transmembrane domain and a cytoplasmic tail. These often interact with other cell surface proteins and are involved in cell signalling. Large, gel-forming mucins (MUC2,5AC, 5B,6) have a Cys-rich D domain and are polymerized to several millions of molecular weight.

## 16. Role of Mucins

The role of mucins varies from protection in mucus to cell adhesion and regulation of tumour survival and diagnostic markers in cancer. Some mucins carry hundreds of O-glycans with different structures and lengths up to more than 20 sugars, which are hydrophilic and can be negatively charged due to sialic acid and sulfate esters and bind to metal ions. The properties of mucins are dictated by the abundance of O-glycans, which cause an extended conformation of mucins and prevent protease cleavage. Mucins play an essential role in intestinal health, supporting beneficial microbiota and avoiding pathogens. O-glycans form adhesive receptors for bacteria and other microbes that can degrade them, use glycans as a source of nutrition, and compete for adhesion [62,63]. O-glycans also play a role in fertilization and sperm–egg binding. Since O-glycans often contain epitopes and antigens, e.g., Lewis-type structures, that are recognized by antibodies and lectins, and they have a controlling function in the immune system. Simple O-glycans are often associated with cancer cells, and sialylated O-glycans have been shown to increase in cancer [21]. They regulate the survival and attachment of cancer cells and can be useful biomarkers and basis for vaccines. Some growth factors and cytokine receptors are O-glycosylated. For example, death receptors require O-glycosylation for efficient apoptotic signalling in tumours [64].

There are four major mucin type O-glycan core structures (1 to 4) (Table 4), with core 1 and 2 being mostly universal, while core 3 and 4 are found in mucins from specific tissues such as intestinal or lung tissue [23]. These core structures can be extended by a variety of sugars and epitopes. Core 5 is a rare structure that is not extended but can be sialylated, and it has been found in human meconium and adenocarcinoma [30,65]. Other core structures (6 to 8) are extremely rare. The core structures are expected to have different conformations and thus display their extensions and epitopes differently. For example, cores 2 and 4 have a GlcNAcβ1-6 branch that can occupy a significant amount of space due to the rotation of the 1-6 bond. This was established by analyzing core 1 to 6 structures using NMR and energy calculations [66].

The overwhelming majority of mucins are bound to the cell membrane and have multiple roles in signalling and recognition events. These Type I membrane glycoproteins can form complexes with growth factors and tyrosine kinases; they can be receptors for mammalian lectins such as Siglecs and can interact with proteins and cells of the immune system, controlling inflammatory responses and immune responses against tumours. The cytoplasmic C-termini can be phosphorylated and interact with proteins of signal transduction and regulation of survival and cell death signals. The extracellular domains can also be cleaved and then secreted as soluble mucins with a number of additional roles. In cancer, MUC1 expression in particular has been found to be elevated in addition to alterations of their O-glycan structures [67]. For example, simple O-glycan structures Tn and T antigens (Table 2) and their sialylated forms are typically present in increased amounts in cancer, together with anti-Tn and anti-T antibodies. MUC1 and MUC4 have consistently been shown to be highly expressed in cancer. Thus, cancer vaccines based on MUC1 have been developed and tested in clinical trials, with some success in diminishing tumour load. MUC4 is a marker for pancreatic cancer and is highly immunogenic, and vaccines have been developed to target cells expressing MUC4. MUC16 is also known as ovarian cancer antigen CA125.

## 17. Glycosyltransferases That Assemble Mucin O-Glycans

O-glycans are assembled step by step in the Golgi and are directly attached to the side chain oxygen of Ser or Thr residues in the completed polypeptide chains. There is no known single amino acid sequence common for all O-glycans, but databases allow an estimate of probable O-glycosylation based on statistics of known sequences around O-glycosylation sites of glycoproteins. The first sugar residue of O-glycans (GalNAc) is added by GTs localized to Golgi membranes (Table 1). GALNT occurs as 20 isoenzymes that O-glycosylate peptides in humans. GalNAc is transferred to peptide by a family of at least 20 polypeptide GalNAc-transferases (GALNTs) that are expressed in a cell-type-specific fashion and support the essential function of mucin O-glycans. GALNTs are classified as GT27 enzymes, and most have a lectin domain connected to the catalytic domain by a flexible linker sequence. GALNTs have similar broad specificities for the amino acids near the O-glycosylation site, with a preference for nearby Pro residues that may enhance recognition of the hydroxyls of Ser/Thr. GALNT7 requires a pre-existing GalNAc-O-glycan in the peptide [68]. The crystal structures of GALNT1,2,3,4,7,10,12 have been determined and show complex structures with a GT-A fold in the GT domain. A DxH motif is found to coordinate UDP-GalNAc and a Mn^2+^ cofactor [69]. Several GALNTs are aberrantly expressed in cancer. For example, the expression and activity of GALNT14 in hepatocellular and gastrointestinal cancer may be a useful disease marker [25].

GalNAcα-Ser/Thr (Tn antigen) as well as its sialylated form sialylα2-6GalNAcα-Ser/Thr (sialyl-Tn antigen) are epitopes commonly found in cancer. The α6-sialylation of GalNAc prevents further processing, with the exception of O-acetylation of Sia, which then masks the sialyl-Tn antigen.

**Figure 4:**
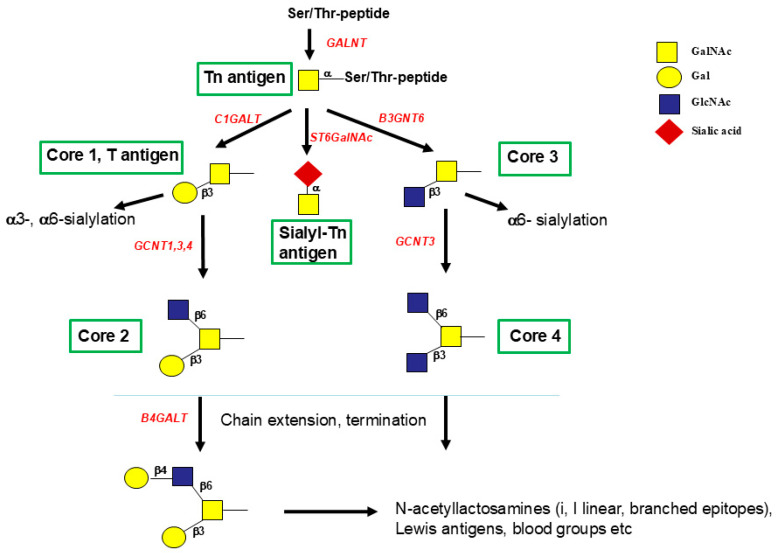
O-glycosylation pathways, synthesis of cores 1 to 4. All mucin-type O-glycans are linked through GalNAcα to Ser or Thr. This linkage is formed by one or more of 20 polypeptide GalNAc-transferases (GALNTs) that are expressed in a cell type-specific fashion. GalNAc can be converted to core 1 by β1,3-Gal-transferase C1GALT1, or to core 3 by β1,3-GlcNAc-transferase B3GNT6. Unmodified GalNAc is recognized as the cancer-associated Tn antigen. If sialic acid is added to GalNAc by ST6GalNAc1, the O-glycan becomes the Sialyl-Tn antigen, which is not further extended. Core 1 can be converted to core 2 by GCNT1, GCNT3, or GCNT4. Core 3 is converted to core 4 only by GCNT3. Unmodified core 1 is named the T antigen, which is often found in cancer. Sialylation of the T antigen also stops further extension to complex chains.

The enzymes that synthesize the four major mucin-type O-glycan core structures (1 to 4) have been characterized and the genes cloned (Figure 4). Crystal structures are available for core 1 β1,3-GalT (C1GALT1) and core 2 (β1,6-GnT, GCNT1). The expression of C1GALT1 is uniquely dependent on the coexpression of the chaperone COSMC [70], which prevents protein degradation by ensuring proper folding of C1GALT1. In the absence of Cosmc, C1GALT1 is ubiquitinated and targeted to the proteasome for degradation. It has been observed that a number of cancer cells lack core 1 and its further metabolites, and cells express the Tn and sialyl-Tn antigen due to the lack of COSMC. The occurrence of unmodified core 1, the T antigen, is common in cancer and during early development as an oncofoetal antigen.

C1GALT1 acts on a variety of GalNAcα-R acceptor substrates, including glycoproteins and the GalNAcα-glycopeptides, synthesized by the Paulsen group. Control assays for C1GALT1 are usually carried out with GalNAcα-Bn acceptor, which is an acceptor for core 1 and core 3 synthesis and has been used as an O-glycosylation inhibitor in cells since it competes with natural glycoprotein biosynthesis. The problem is that GalNAcα-Bn is converted to a number of benzyl-terminating hydrophobic O-glycan structures, which overwhelm the storage and transport capacity of the cell and induce apoptotic cell death [71]. Since core 1 and 3 are precursors for core 2 and 4 structures (Figure 4) GalNAcα-Bn inhibits the synthesis of core 1 to 4 in glycoproteins.

Derivatives of GalNAcα-Bn were synthesized and tested as potential substrates and inhibitors [72]. Removal or substitution of the 6-hydroxyl of GalNAc slightly reduced C1GALT1 activity. 6-O-(4,4-azo)pentyl- GalNAcα-Bn was photosensitive and inhibited Gal incorporation into GalNAcα-Bn (Table 3). The results indicate that the enzyme has a relatively broad acceptor specificity and does not require the 6-hydroxyl of GalNAc, but it needs the 3- and axial 4-hydroxyl as essential requirements for binding and activity.

Yeh et al. [73] cloned a number of β1,6-GnTs, GCNT1, 2 and 3. GCNT1 introduces a GlcNAcβ1-6 branch onto core 1, Galβ1-3GalNAc-R, linked to various hydrophobic groups or peptides, and synthesizes core 2 (Figure 4). This activity increases in many cancer and leukaemia cells [74] and upon cancer cell differentiation. GCNT1 plays an important role in the immune system by providing the scaffold for selectin ligands sialyl-Lewis x, crucial for lymphocyte interactions [75]. In specific breast cancer cells, however, GCNT1 is not expressed [76]. The expression shows large variations among cancer cells derived from prostate cancer [31]. Although the enzyme is an inverting GT with a GT-A fold [33], it does not require Mn^2+^ as a cofactor. GCNT1 is N-glycosylated and requires several conserved Cys residues for the formation of disulfide bonds. Amino acid reagents that target non-specifically His, Trp, and Cys inhibited purified GCNT1 activity up to 100%.

The groups of Hans Paulsen and Khushi Matta synthesized many derivatives of core 1, which revealed the specificity of GCNT1. Galβ1-3(6-deoxy) GalNAcα-Bn was found to be a weak inhibitor. Surprisingly, the excellent substrate Galβ1-3GalNAcα-p-nitrophenyl inhibited GCNT1 activity from acute myelogenous leukaemia cells by 87% upon UV irradiation.

Another β1,6-GnT, GCNT4, has similar activity and 44% sequence identity with GCNT1 but has a selected tissue expression. It is associated with the thymus and downregulated in a number of different cancer cells and tissues [26].

The metal ion-independent β1,6-GnT, GCNT3, synthesizes both core 2 from core 1 and core 4 from core 3. The enzyme has high activity in colonic tissue, explaining the abundance of core 4 O-glycans in colonic mucins [23]. GCNT3 has a 50.35% sequence identity with GCNT1 and is also classified as GT14, but shows very different inhibition by bis-imidazolium salts.

In many cancer cells, both core 3 and 4 synthesis by B3GNT6 and GCNT3 showed altered activity [77]. GCNT3 is not expressed in normal or cancer-derived prostate cells [31], but is variably expressed in other cancer types [29]. Huang et al. [78] suggested a role for GCNT3 in suppressing cancer.

The crystal structure of murine GCNT1 [79,80] suggests that the DxD motif is replaced by the DE sequence, with Glu320 as the catalytic base. Two positively charged amino acids (Arg378 and Lys 401) take the role of a Mn^2+^ ion in stabilizing the UDP leaving group. These amino acids are conserved among these β1,6-GnTs. The other metal ion-independent β1,6-GnT, GCNT2, is 37.8% identical to GCNT1 but has a different acceptor specificity and synthesizes the branch of the I antigen [73]. It is not clear which of the Arg residues cooperate with Lys in binding UDP.

GalNAcα-Bn is also an acceptor for β1,3-GnT, B3GNT6, that synthesize core 3. The enzyme was discovered in rat and human colon tissues that are rich in mucins carrying core 3. Many colon cancer cell lines and colon cancer tissue, however, have very low enzyme activity [77]. While GnTs I, II, and V are variably active in normal and cancer prostate cells, B3GNT6 was not detected [31]. The lack of core 3 synthesis may be one factor promoting core 1 (T antigen) expression.

Rare core structures in human glycoproteins include cores 5 to 8 (Table 4). Core 5, GalNAcα1,3-GalNAc-, was found in human intestinal fetal mucins and in adenocarcinoma [30,65]. A preliminary report of core 5 synthesis using the GalNAc-mucin acceptor [81] showed that Sia may be added to GalNAc after the formation of core 5. However, the gene of this α1,3-GalNAcT has not yet been identified. It is possible that this activity is caused by a variant of a known enzyme. In contrast to O-glycan core structures 1 to 4, core 5 has not been found to be elongated, and its role is unknown. The activities or genes encoding the GTs that synthesize core 6 to 8 structures remain to be identified. It is possible that core 6 in mucins is a degradation product of core 2, due to bacterial hydrolases or due to degradation during O-glycan isolation.

## 18. Elongation of O-Glycans

A wide array of glycosyltransferases can extend core structures in complex pathways and create hundreds of different O-glycan structures with lengths of more than 20 sugar residues in mucins. The Gal and GlcNAc residues of O-glycan core structures can be elongated by enzymes that similarly elongate N-glycans. Very few of these enzymes are specific to O-glycans. This includes β1,3-GnT, B3GNT3, which adds GlcNAc to Gal of core 1 and 2 [23]. The enzyme can act on mucins or hydrophobic acceptors with core 1 or 2 structures and thus allows the attachment of complex glycans and lymphocyte homing receptors [82]. Core 2 sometimes carries an unusual GlcNAcα1-4 residue linked to Gal in gastric cells, such as pyloric glands and neck cells [83]. The retaining α1,4-GnT A4GNT may use the large mucin MUC6 as a scaffold. The expression of A4GNT together with MUC6 was found to suppress the proliferation of pancreatic tumour cells [84].

Several SiaTs are specific for mucin-type O-glycans and generally block further extension. All human SiaTs are classified in the inverting GT29 family and have common sialylmotifs that contribute to the binding of substrates [85]. ST6GalNAc1-4 transfer Sia from CMP-βSia in α2-6 linkage to GalNAc-mucins with different specificities for core 1 and sialylated core 1. ST6GalNAc1 primarily synthesizes the sialyl-Tn antigen, while ST6GalNAc2 primarily synthesizes the sialyl-T antigen. ST6GalNAc2 can act on GalNAc- and core 1- or 3-peptide acceptors [28]. The crystal structure of ST6GalNAc2 with CMP [86] shows a GT-A variant2 fold [87]. ST6GalNAc3 and 4 utilize core 1 substrates that have already been sialylated by ST3GAL1, but they do not require a peptide in the acceptor. ST6GalNAc3 has a restricted expression mainly in the brain and kidney.

ST3Gal1 specifically adds Sia in α2-3 linkage to Gal of core 1 and 2 linked to peptide or a hydrophobic group. The ST3GAL1 product, Sialyl-T antigen, is a common antigen in cancer cells. ST3Gal1 is overexpressed in breast cancer tissues [27] and in prostate cancer cells [31], where it controls proliferation, migration, and apoptosis. Porcine ST3Gal1 has been crystallized and revealed a single Rossmann domain, like other SiaTs, and a β-sheet core that contributes to binding CMP-Sia and His319 as a possible catalytic residue [88].

## 19. Synthesis of Glycopeptides

Because of the importance of mucins and O-glycosylation in human health and diseases such as cancer, the Paulsen group synthesized more than 200 peptides and glycopeptides derived from MUC1 to 4 and other glycoproteins for studies of their biological role and pathways of O-glycan biosynthesis (Table 4). It has been extremely difficult to determine the sites of O-glycan addition in complex mucins and to assign O-glycan structures to specific Thr and Ser residues. Thus, peptides and glycopeptides have been a tremendous tool to discover mechanisms of O-glycosylation, the influence of amino acids near O-glycosylation sites in acceptor substrates for GALNTs, and new cancer-associated and immunogenic epitopes.

Hundreds of glycopeptides have been produced by Paulsen’s group, together with the unglycosylated control peptides as standards and controls. The development of solid-phase synthesis of GalNAcα- and Galβ1-3GalNAcα-glycopeptides using protected glycosylamino acids was a breakthrough [89,90]. Novel glycosyl amino acids were synthesized as building blocks for Fmoc-based continuous-flow synthesis using multi-column technology. This achieved synthesis of several glycopeptides simultaneously without significant degradation of the glycan-peptide bond and beta-elimination of the O-glycan. The amino acid sequences had different lengths up to 23 amino acids and were based on those from the VNTR of MUC1, 2, 3, or 4. The glycans attached to specific Thr or Ser residues included one or more GalNAc residues and selected core 1 to 7 structures (Table 4). Glycopeptides were purified, and their sequences and glycosylation were confirmed by HPLC, MS, and NMR. Computational analyses and NMR determined the conformations of glycopeptides that could potentially be recognized by individual enzymes.

To examine the preferred attachment sites for GALNTs, a series of glycopeptides were synthesized that were overall neutral with an acetyl group at the N-terminus and amide at the C-terminus and contained one or more GalNAcα-Thr/Ser or Galβ1-3GalNAcα, or in the unnatural β-linkage to Thr. The glycopeptide reaction products were analyzed by the amount of radioactive sugar transferred and by their HPLC elution times using standard glycopeptides.

Within a series, the positions of glycans and some of the Thr/Ser residues were altered in a systematic fashion to include all possibilities of glycan attachments [91]. In addition, Pro and Thr were replaced by other amino acids at +1 or -1 positions to determine their role in a GALNT substrate. The smallest peptide used as a positive control was Ac-TPPP, which showed the highest activity as an acceptor for highly purified bovine GALNT1 [92,93]. The small acceptor substrate does not have a C-terminal protection group, but clearly has easy access to the catalytic site. In glycopeptides 8 amino acids long, Pro in the +3 position (towards the C-terminus) supported the activity. None of the Thr residues are exclusive sites for GalNAc addition, but the amino acid sequences and sites of existing glycosylation clearly dictate further O-glycosylation. Substrates with core 1 in β-linkage are less active, likely due to the different conformations compared to the natural α-linkage.

A series of different lengths and GalNAc attachment to Ser did not show significant activity. It is possible that the slight hydrophobic effect of Thr plays a role in enzyme recognition. However, other members of the GALNT family may show different results and could act on Ser. Generally, site specificity is generally broad, and larger glycopeptides and those with different amino acid sequences remain to be tested as models for mucin synthesis. Glycosylated peptides are more rigid in their conformations compared to peptide alone, and large O-glycans cause crowding. Non-mucin glycoproteins often have glycosylation sites only near the N-terminus, and protein folding may block glycosylation. Mucins have large complex O-glycans, often at adjacent amino acids, and the processing enzymes only have access to their substrates in an unfolded (bottlebrush) conformation of the glycoprotein.

Although there is clearly no consensus sequence for O-glycosylation, TxxP is one of the preferred sites for mucin O-glycosylation by GALNT1. O-glycosylation is extremely complex, with substrate conformation, composition, hydrophobicity, existing glycosylation, as well as overall structure controlling recognition by GALNTs and other GTs.

Subsequently, C1GalT1, GCNT1, and B4GALT1 were examined for their ability to use glycopeptide substrates in a site-specific manner [93,94,95]. The synthesis of core 1 by partially purified rat liver C1GALT1 was examined with GalNAc-containing glycopeptides containing 1 to 3 GalNAc-Thr. Compared to the GalNAcα-Bn acceptor, all of the GalNAc-glycopeptides were good substrates for the enzyme that could add 1 to 3 Gal residues to each GalNAc. The enzyme was strongly influenced by the overall composition and especially amino acids at the -1 and +1 positions. These studies indicate that in addition to initial glycosylation, the second step in the glycosylation pathways of O-glycans is also controlled by the structure and glycosylation of the peptide core of substrates.

The activity of purified human GCNT1 was influenced by amino acid composition, the position of the core 1 substrate, and other glycans within the glycopeptide. Surprisingly, glycopeptides with core 1 in the unnatural β-linkage were also variably good substrates for GCNT1. The extension of core 2-glycopeptides with purified bovine B4GALT1 also showed significant variability. However, B4GALT1 has a higher activity with GlcNAcβ-Bn as a substrate than with glycopeptides.

Overall, glycopeptides helped to determine that the control of O-glycosylation is based on a multitude of complex factors, including amino acid position, properties, and charge as well as existing glycosylation. GTs that act close to the peptide (GALNT and C1GALT1) are more specific than those adding the third (GCNT1) or fourth (B4GALT1) sugar residue. Currently, Paulsen glycopeptides are still under investigation for their ability to form GT substrates, to discover novel enzymes, to enzymatically synthesize novel glycopeptides, and as potential inhibitors of bacterial biofilm formation.

## 20. Conclusions

**Figure 5:**
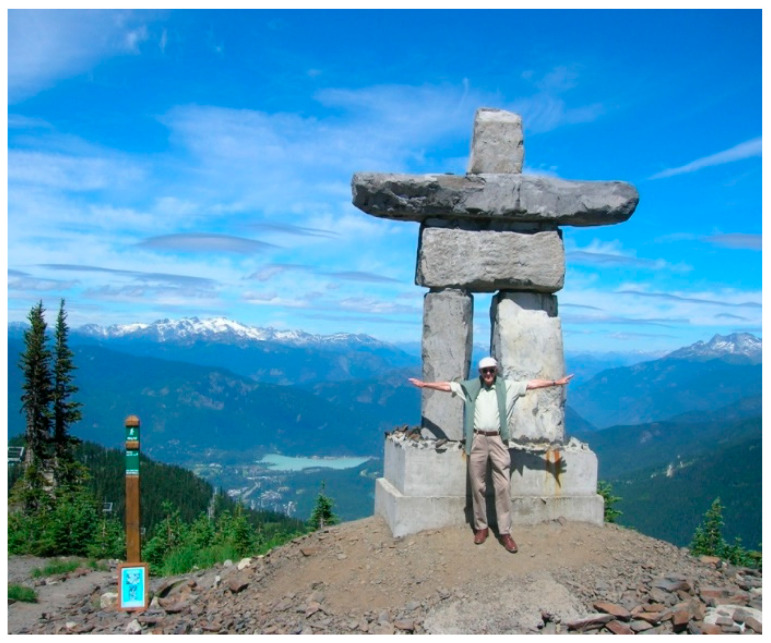
Hans Paulsen at the International Carbohydrate Symposium in Whistler, Canada (2006).

Paulsen developed new synthetic technologies by synthesizing complex large N-glycans, modified glycans, and their fragments. This has been documented in hundreds of publications and presentations (Figure 5). His new synthesis included the discovery of methods to synthesize substituted Manβ-R compounds and to initiate solid-phase glycopeptide synthesis. Paulsen’s substrates are essential for the discovery of new enzymes, for defining enzyme specificities that control the pathways of N- and O-glycosylation, and for defining the biological roles of glycans. The roles include receptors for lectins and adhesion proteins in eukaryotic systems and in bacteria that use mucins as a niche for survival and nutrition. Disease-specific alterations in GTs could be discovered. The inhibitors that Paulsen synthesized will help in the development of future therapies for cancer and other diseases, where the reduction in complex glycans could be beneficial. Many synthetic routes and analytical tools for complex glycans and glycopeptides have been developed and subsequently adopted worldwide. Future studies are expected to use this information to understand how enzymes recognize their glycan substrates and how this influences pathways of glycoprotein biosynthesis. We are grateful for the hard work of Hans Paulsen and his coworkers and students. He believed that the principles behind the contributions to glycobiology are based on the enjoyment of chemistry!

## Figures and Tables

**Table 1:** List of major human glycosyltransferases and glycosidases involved in glycoprotein biosynthesis. The GT names and accession numbers are derived from the Uniprot database. Included here are also the major glycoside hydrolases (GHs glucosidases and mannosidases) that are essential for the maturing of N-glycans in the ER and Golgi. *GTs with known crystal structure from human or animal sources; GALNT1,2,3,4,7,10,12 have been crystalized. CAZy GT, Carbohydrate Active Enzyme database classification; Dol, dolichol; GnT, GlcNAc-transferase; P, phosphate; Sia, sialyl; T, transferase. GT Mechanisms and Folds, and CAZy GT classification: Inverting (11,14,16,17,18,29,31,54), Inverting GT-A (2,7,12,13), Inverting GT-B (1,10,23,33), Inverting GT-C (22,57,58,59), Retaining (32), Retaining GT-A (6,27), Retaining GT-B (4).

Enzyme Name	Uniprot	CAZy GT	Donor	Product
ER: N-glycan biosynthesis				
*GlcNAc-P-T DPAGT	Q9H3H5	-	UDP-GlcNAc	GlcNAc-PP-Dol
β4GlcNAcT ALG13/14	Q9NP73	1	UDP-GlcNAc	GlcNAc_2_-PP-Dol
	Q96F25			
DPM1	O60762	2	GDP-Man	Manβ-P-Dol
β4ManT ALG1	Q9BT22	33	GDP-Man	Man-GlcNAc_2_-PP-Dol
α3/6ManT ALG2	Q9H553	4	GDP-Man	Man_3_-GlcNAc_2_-PP-Dol
α2ManT ALG11	Q2TAA5	4	GDP-Man	Man_5_-GlcNAc_2_-PP-Dol
α3ManT ALG3	Q92685	58	Dol-P-Man	Man_7_-GlcNAc_2_-PP-Dol
α2ManT ALG9	Q9H6U8	22	Dol-P-Man	Man_9_-GlcNAc_2_-PP-Dol
α2ManT ALG12	Q9BV10	22	Dol-P-Man	Man_9_-GlcNAc_2_-PP-Dol
DolPGlc synthase ALG5	Q9Y673	2	UDP-Glc	Glcβ-P-Dol
α3GlcT ALG6	Q9Y672	57	Dol-P-Glc	Glc-Man_9_-GlcNAc_2_-Dol
α3GlcT ALG8	Q9BVK2	57	Dol-P-Glc	Glc_2_-Man_9_-GlcNAc_2_-Dol
α2GlcT ALG10	Q5BKT4	59	Dol-P-Glc	Glc_3_-Man_9_-GlcNAc_2_-Dol
OST complex	-	-	Glycan-Dol	Glycan-Asn
α2Glc MOGS	Q13724	GH63		Glc_2_-Man_9_-GlcNAc_2_-Asn
α3Glc GANAB	Q14697	GH31		Man_9_-GlcNAc_2_-Asn
α2Man MAN1B1	Q9UKM7	GH47		Man_8_-GlcNAc_2_-Asn
Golgi: N-glycan processing				
α2Man MAN1A1	P33908	GH47		Man_5_GlcNAc_2_-Asn
*β2GnT I MGAT1	P26572	13	UDP-GlcNAc	GlcNAcβ2Man_5_GlcNAc_2_-
α3,6Man MAN2A1	Q16706	GH38		GlcNAcβ2-Man_3_GlcNAc_2_-
*β2GnT II MGAT2	Q10469	16	UDP-GlcNAc	GlcNAcβ2_2_Man_3_GlcNAc_2_-
β4GnT III MGAT3	Q09327	17	UDP-GlcNAc	bisecting GlcNAcβ4Manβ-
β4GnT IV MGAT4A	Q9UM21	54	UDP-GlcNAc	GlcNAcβ_3_Man_3_GlcNAc_2_-
*β6GnT V MGAT5	Q09328	18	UDP-GlcNAc	GlcNAcβ_4_Man_3_GlcNAc_2_-
*GNPTAB	Q3T906	-	UDP-GlcNAc	GlcNAc-6P-Man
GNPTG complex	Q9UJJ9	-	UDP-GlcNAc	GlcNAc-6P-Man
*α6FucT FUT8	Q9BYC5	23	GDP-Fuc	N-glycan core Fuc
*α6SiaT ST6Gal1	P15907	29	CMP-Sia	complex N-glycans
α8SiaT ST8SIA2	Q92186	29	CMP-Sia	Siaα2-8Sia-
*α8SiaT ST8SIA3	O43173	29	CMP-Sia	Siaα2-8Sia-
α8SiaT ST8SIA4	Q92187	29	CMP-Sia	Siaα2-8Sia-
Golgi: O-glycan biosynthesis				
*GALNT1	Q10472	27	UDP-GalNAc	O-glycan initiation
*β3GalT C1GALT1	Q9NS00	31	UDP-Gal	core 1
*β6GnT GCNT1	Q02742	14	UDP-GlcNAc	core 2
β6GnT GCNT3	O95395	14	UDP-GlcNAc	core 2/4
β6GnT GCNT4	Q9P109	14	UDP-GlcNAc	core2
β3GnT B3GNT3	Q9Y2A9	14	UDP-GlcNAc	core 1,2 elongation
β3GnT B3GNT6	Q6ZMB0	31	UDP-GlcNAc	core 3
α4GnT A4GNT	Q9UNA3	32	UDP-GlcNAc	GlcNAcα4-Gal of core 2
*α3SiaT ST3Gal1	Q11201	29	CMP-Sia	Sia-core 1
α6SiaT ST6GalNAc1	K7EMB6	29	CMP-Sia	Sia-GalNAc-Thr
*α6SiaT ST6GalNAc2	Q9UJ37	29	CMP-Sia	Sia-GalNAc/Gal-GalNAc-Thr
α6SiaT ST6GalNAc3	Q8NDV1	29	CMP-Sia	Sia-Gal-(Sia)-GalNAc)
α6SiaT ST6GalNAc4	Q9H4F1	29	CMP-Sia	Sia-Gal (Sia)-GalNAc
Golgi: N- and O-glycan chain extension and termination				
*iβ3GnT B3GNT2	Q9NY97	31	UDP-GlcNAc	i antigen
β3GnT B3GN8	Q7Z7M8	31	UDP-GlcNAc	i antigen
β6GnT GCNT2	Q8N0V5	14	UDP-GlcNAc	I antigen
β4GalNAcT B4GALNT3	Q6L9W6	7	UDP-GalNAc	Lac-diNAc
*α3GalNAcT, GTA, BGAT	P16442	6	UDP-GalNAc	Blood group A
	V5ZDP0			
β4GalNAcT B4GALNT2	Q8NHY0	12	UDP-GalNAc	Sda antigen
*α3GalT GTB	V9GWR7	6	UDP-Gal	Blood group B
*β4GalT B4GALT1	P15291	7	UDP-Gal	GlcNAc extension
β4GalT B4GALT2	O60909	7	UDP-Gal	GlcNAc extension
β4GalT B4GALT3	O60512	7	UDP-Gal	GlcNAc extension
*β3GalT B3GALT5	Q9Y2C3	31	UDP-Gal	GlcNAc extension
α2FucT FUT1	P19526	11	GDP-Fuc	Blood group H/O
α2FucT FUT2	Q10981	11	GDP-Fuc	Blood group H/O
α3/4FucT FUT3	P21217	10	GDP-Fuc	Lewis antigens
α3FucT FUT4	P22083	10	GDP-Fuc	Lewis antigens, type 2 chains
α3/4FucT FUT5	Q11128	10	GDP-Fuc	Lewis antigens
α3FucT FUT7	Q11130	10	GDP-Fuc	sialyl-Lewis
*α3FucT FUT9	Q9Y231	10	GDP-Fuc	Lewis antigens
α3SiaT ST3Gal3	Q11203	29	CMP-Sia	Sia-Gal-GlcNAc-
α3SiaT ST3Gal4	Q11206	29	CMP-Sia	Sia-core 1/Gal-GlcNAc
α3SiaT ST3Gal6	Q9Y274	29	CMP-Sia	Sia-Gal-GlcNAc
α8SiaT ST8SIA6	P61647	29	CMP-Sia	Siaα2-8Sia

**Table 2:** List of mucin core structures and epitopes of both O- and N-glycans. Human glycoproteins have O-glycan structures initiated with GalNAc or core structures 1 to 4. Other core structures (5 to 8) are rare. Based on the variable tandem repeat sequences of MUC1, MUC2, MUC3, and MUC4, Paulsen’s group synthesized hundreds of glycopeptides carrying one or more O-glycans with core 1 to 7. The occurrences of most of these structures have been found to be altered (increased or decreased) in cancer [20,21,22,23,24,25,26,27,28,29,30,31].

Epitope	Structure	Alteration in Cancer
Tn antigen	GalNAcα-Ser/Thr-	Increased
Sialyl-Tn antigen	Siaα2-6GalNAcα-	Increased
Core 1, T antigen	Galβ1-3GalNAcα-	Increased
Sialyl-T antigen	Siaα2-3Galβ1-3GalNAcα-	Increased
Core 2	GlcNAcβ1-6(Galβ1-3)GalNAcα-	Variable
Core 3	GlcNAcβ1-3GalNAcα-	Decreased
Core 4	GlcNAcβ1-6(GlcNAcβ1-3)GalNAcα-	Decreased
Core 5	GalNAcα1-3GalNAcα-	Increased
Core 6	GlcNAcβ1-6GalNAcα-	
Core 7	GalNAcα1-6GalNAc-	
Core 8	Galα1-3GalNAc-	
Sialyl-Lewis x	Siaα2-3Galβ1-4(Fucα1-3)GlcNAcβ-	Increased
Sda/Cad	GalNAcβ1-4(Siaα2-3)Galβ-	Decreased
i antigen	Galβ1-4GlcNAcβ1-3Galβ1-	Variable
I antigen	Galβ1-4GlcNAcβ1-6	
	(Galβ1-4GlcNAcβ1-3)Galβ1-	
LacdiNAc	GalNAcβ1-4GlcNAcβ1-3-	
Sialyl-LacNAc	Siaα2,6Galβ1-4GlcNAc	Increased
Blood group O(H)	Fucα1-2Galβ-	
Blood group A	GalNAcα1-3(Fucα1-2)Galβ-	
Blood group B	Galα1-3(Fucα1-2)Galβ-	
Complex N-glycans	Highly branched N-glycans	Increased GnT V
Sialylated termini	Sialylated N-glycans	Increased ST6Gal1

**Table 3:** Acceptor substrate analog inhibitors for glycosyltransferases. Paulsen’s group synthesized many acceptor substrates that are analogs of the natural N-glycans found in humans. Using these compounds, the activities of several glycosyltransferases were determined, and inhibitors were found. The compounds listed in this table showed significant inhibition of GT activity. GlcNAc-naphthyl derivatives were synthesized by W.A. Szarek, Queen’s University. oct, octyl; *p*np, *p*-nitrophenyl; -R, various hydrophobic groups.

Enzyme	Inhibitors
GnT I	Manα6(Manα3)4-O-methyl-Manβ4GlcNAc
	Manα6(6-O-methyl-Manα3)Manβ-oct
	Manα6(6-O-4,5-epoxy-pentyl-Manα3)Manβ-oct
	Manα6(6-O-4,4-azo-pentyl-Manα3)Manβ-oct
GnT II	2-deoxy-Manα6(GlcNAcβ2Manα3)Manβ-oct
	GlcNAcβ2Manα3Manβ-oct
	3-O(4,4 azo)pentylManα6(GlcNAcβ2Manα3)Manβ-oct
	2-deoxy-Manα6(GlcNAcβ2Manα3)Manβ-oct
GnT V	GlcNAcβ2(6-deoxy)Manα6Glcβ-O-R
	GlcNAcβ2(4-O-methyl)Manα6Glcβ-O-R
	GlcNAcβ2(6-deoxy, 4-O-methyl)Manα6 Glcβ-O-R
	GlcNAcβ2(4,6-di-O-methyl)Manα6Glcβ-*p*np
O-glycan synthesis	GalNAcα-aryl
C1GALT1	6-O-(4,4-azo)pentyl-GalNAcα-Bn + UV
GCNT1	Galβ3GalNAcα-*p*np + UV
	Galβ3(6-deoxy) GalNAcα-Bn
β4GalT	GlcNAcβ-naphthyl derivatives
	1-thio-N-butyryl-GlcNAcβ-2-naphthyl

**Table 4:** Glycopeptides synthesized by Hans Paulsen. Paulsen synthesized hundreds of glycopeptides, some with 1 to 23 amino acids and 1 to 3 glycan chains attached to the longer peptides. Glycans were various combinations of GalNAc, core 1 to 7. The table shows one example of each series and the systematic changes to make variants of either amino acids, attachment site of glycan, type of core structure, or number of glycans attached. Bold amino acids carry glycans.

Different Series with Examples of Glycopeptides	Variants Synthesized
AcTPPP	
1. AP**T**^GalNAcα^ S^GalNAcα^ SS	Thr/Ser, position of GalNAc
2. AP**T**^Galβ3GalNAcα^ SSS	Thr/Ser, position of core 1
3. Ac-**T**^GalNAcα^ P-*t*-Bu	protected N/C-terminal protection
4. AP**T**^GalNAcα^-SSSTKKT	peptide length
5. Ac-V**T**^GalNAcα^ P-NH_2_	peptide length, N/C-terminal protection
6. Ac-PTT**T**^GalNAcα^ PIST-NH_2_	amino acids, position of GalNAc
7. Ac-PTPTGTQTPT**T**^GalNAcα^ TPITTTTTVTPT-NH_2_	number of GalNAc
8. AHGVT**S**^Galβ3GalNAcα^ APDTRPAPGSTAP **T**^Galβ3GalNAcα^ A	amino acids, position and number of GalNAc, core 1
9. PTTTPITTTG	amino acid sequence
10. Ac-P**S**^GalNAcα^ **S**^GalNAcα^ **S**^GalNAcα^ PIST-NH_2_	amino acids, number, Ser/Thr position of glycans
11. TT**T** ^GlcNAcβ3GalNAcα^ VTP**T** ^GlcNAcβ3GalNAcα^ PTG	number and position of GalNAc, core 3
12. TETTSHS**T**^GalNAcα^ PG	number and position of GalNAc, core 3, length of peptide
13. TT**T**^Galβ3GalNAcα^ VTP**T** ^Galβ3GalNAcα^ PTG	position and number of core 1
14.TTTVTPTP**T**^GlcNAcβ6(Galβ3)GalNAcα^G	position and number of core 1, 2
15. TT**T** ^GlcNAcβ6(Galβ3)GalNAcα^ VTP**T**^Galβ3GalNAcα^ PTG	position of core 1, 2
16. TT**T**^GlcNAcβ6(GlcNAcβ3)GalNAcα^ VTP **T**^GlcNAcβ6(GlcNAcβ3)GalNAcα^ PTG	position and number of core 4
17. TTTVTP**T**^GlcNAcβ6GalNAcα^ PTG	position and number of core 6
18. TT**T**^GlcNAcβ6(GlcNAcβ3)GalNAcα^ VTP**T**^GlcNAcβ6GalNAcα^ PTG	position and number of core 4, 6
19. Ac-P**T**^GalNAcα^ **T**^Galβ3GalNAcα^ **T**^Galβ3GalNAcα^ PIST-NH_2_	position and number of GalNAc and core 1
20. Ac-PTT**T**^Galβ3GalNAcα^ PIST-NH_2_	position of core 1α or β, amino acids
21. TETTSHS**T**^Galβ3GalNAcα^ PG	position and number of core 1, 2, 4, 6
22. Ac-ELS**T**^GalNAcα^ **T** ^GalNAcα3GalNAcα^ GPG- NH_2_	amino acids, number and position of GalNAc, core 5, 7
23. Ac-ELA**T**^GalNAcα^ VGPG-NH_2_	amino acids, GalNAc, core 5, 7
